# Exacerbation of allergic inflammation in mice exposed to diesel exhaust particles prior to viral infection

**DOI:** 10.1186/1743-8977-6-22

**Published:** 2009-08-14

**Authors:** Ilona Jaspers, Patricia A Sheridan, Wenli Zhang, Luisa E Brighton, Kelly D Chason, Xiaoyang Hua, Stephen L Tilley

**Affiliations:** 1 Department of Pediatrics, School of Medicine, University of North Carolina at Chapel Hill, Chapel Hill, North Carolina, USA; 2 Center for Environmental Medicine, Asthma, and Lung Biology, School of Medicine, University of North Carolina at Chapel Hill, Chapel Hill, North Carolina, USA; 3 Department of Nutrition, Gillings School of Global Public Health, University of North Carolina at Chapel Hill, Chapel Hill, North Carolina, USA; 4 Department of Medicine, Division of Pulmonary and Critical Care Medicine, School of Medicine, University of North Carolina at Chapel Hill, Chapel Hill, North Carolina, USA

## Abstract

**Background:**

Viral infections and exposure to oxidant air pollutants are two of the most important inducers of asthma exacerbation. Our previous studies have demonstrated that exposure to diesel exhaust increases the susceptibility to influenza virus infections both in epithelial cells *in vitro *and in mice *in vivo*. Therefore, we examined whether in the setting of allergic asthma, exposure to oxidant air pollutants enhances the susceptibility to respiratory virus infections, which in turn leads to increased virus-induced exacerbation of asthma. Ovalbumin-sensitized (OVA) male C57BL/6 mice were instilled with diesel exhaust particles (DEP) or saline and 24 hours later infected with influenza A/PR/8. Animals were sacrificed 24 hours post-infection and analyzed for markers of lung injury, allergic inflammation, and pro-inflammatory cytokine production.

**Results:**

Exposure to DEP or infection with influenza alone had no significant effects on markers of injury or allergic inflammation. However, OVA-sensitized mice that were exposed to DEP and subsequently infected with influenza showed increased levels of eosinophils in lung lavage and tissue. In addition Th2-type cytokines, such as IL-4 and IL-13, and markers of eosinophil chemotaxis, such as CCL11 and CCR3, were increased in OVA-sensitized mice exposed to DEP prior to infection with influenza. These mice also showed increased levels of IL-1α, but not IL-10, RANTES, and MCP-1 in lung homogenates.

**Conclusion:**

These data suggest that in the setting of allergic asthma, exposure to diesel exhaust could enhance virus-induced exacerbation of allergic inflammation.

## Background

The prevalence of allergic diseases, such as asthma, continues to be on the rise in developed countries. While genetic components most certainly account for some of the susceptibility of developing asthma, the increased prevalence of allergic airway diseases cannot be completely explained on the basis of genetics. A number of extrinsic factors, including nutrition, exposure to environmental pollutants, and lack of exposure to a variety of pathogens during childhood are also suspected to contribute to the susceptibility of developing asthma later on in life as well as the severity of the disease [1]. Moreover, there are a number of triggers that can exacerbate the disease and lead to acute allergic inflammation of the airways. Epidemiologic studies have repeatedly demonstrated that exposure to particulate air pollutants is associated with exacerbation of asthma and increased medication use [2,3]. While there have been a number of very interesting findings regarding potential roles for chronic exposure to diesel exhaust (DE), such as acting as an adjuvant or enhancing the development of allergic airway disease [4-6], it is not yet clear how acute exposure to DE could lead to exacerbation of asthma. In a number of murine models of allergic asthma, exposure to DEP increased airway obstruction and enhanced allergen-dependent and independent airway responsiveness [7-9]. However, human studies examining inflammatory markers in the airways of asthmatics and non-asthmatics have demonstrated that short-term exposure to DE does not worsen pre-existing allergic airway inflammation [10]. It has been suggested that exposure to DE lowers the immune activation threshold for inducing asthmatic symptoms, and therefore increases the susceptibility to exacerbation of the disease [11]. For example, bronchial epithelial cells obtained from asthmatics constitutively expressed higher levels of IL-8, GM-CSF, RANTES, and sICAM and were more sensitive to DEP-induced inflammatory mediator production as compared to non-asthmatic controls [12,13]. In addition, DEP enhanced the activation of T cells obtained from asthmatics, but not from non-asthmatic controls [11].

While exposure to air pollutants such as DE can exacerbate asthma, the majority of clinically documented asthma exacerbations are associated with viral infections [14]. Specifically, rhinovirus (RV) is the most common virus detected in asthmatics during exacerbations, but the spectrum of viruses also includes influenza virus, especially during epidemics [14,15]. Viral respiratory tract infection-induced acute exacerbations of asthma lead to increased health care utilization including hospitalization [16]. In a series of adults hospitalized with asthma, influenza was reported to make up 60% of cases in which a respiratory pathogen was isolated using tissue culture methods [17]. Thus, while not as common as RV in asthma exacerbations, influenza may account for a relatively high proportion of severe cases, and is therefore relevant from clinical and public health perspectives. The mechanisms mediating virus-induced exacerbation of asthma are derived from a number of *in vitro *models as well as experimental human infections with rhinovirus [18]. In addition, a number of animal models have been developed, including models examining the role of influenza in virus-induced asthma exacerbation [19]. The phenotype of virus-induced aggravation of allergic asthma includes increased eosinophilia, increased Th2-type immune response, and increase airway hyperreactivity [19].

Our previous studies have demonstrated that exposure to DE increases the susceptibility to influenza virus infections both in epithelial cells *in vitro *and in mice *in vivo *and that this response is mediated by oxidative stress [20,21]. Recent epidemiologic evidence suggests that personal exposure to oxidant air pollutants increases the severity of virus-induced asthma exacerbations [22]. These observations suggest an additive or synergistic interaction between oxidant air pollution exposure and virus-induced exacerbation of asthma. The study described here investigated whether and how acute exposure to DEP prior to infection with influenza virus affects exacerbation of allergic inflammation in a murine model of asthma. Our results demonstrate that in our model exposure to DEP prior to infection with influenza resulted in significant enhancement of allergic airways inflammation, while either treatment alone did not.

## Results

### Exposure to DEP prior to infection with influenza increases markers of inflammation and lung injury

**Figure 1:**
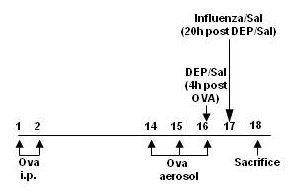
**Schematic of experimental protocol, indicating times of OVA challenge, DEP-exposure, and virus infection**.

**Figure 2:**
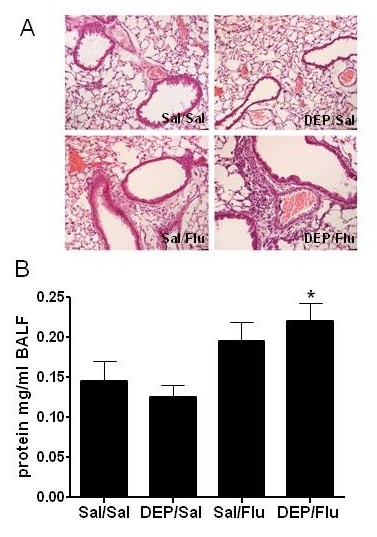
**Analysis of lung injury**. A.) Histochemical analysis of lungs from OVA-sensitized mice exposed to DEP and/or influenza A 24 hours post-infection. Pictures illustrate representative hematoxylin-and eosin (H&E)-stained lung sections. Mice exposed to DEP and infected with influenza A displayed increased cellular infiltration around the blood vessels and airways. B.) Protein levels in BALF obtained from OVA-sensitized mice exposed to DEP and/or influenza A 24 hours post-infection. *significantly different from Sal/Sal group; p < 0.05

C57BL/6 male mice were sensitized and challenged with OVA, then challenged with Influenza (Flu), DEP, or saline as shown in Fig. 1. Histological examination of overall inflammation and infiltration of inflammatory cells was performed, which demonstrated that accumulation of inflammatory cells was greatest in the DEP/Flu group of animals (Fig. 2A). In addition, levels of protein in the BAL fluid (BALF), a marker of edema, were measured. Fig. 2B shows that exposure to DEP prior to infection with influenza significantly increased BALF protein levels. Neither exposure to DEP or infection with influenza alone significantly increased BALF protein.

### Exposure to DEP prior to infection with influenza increases the number of eosinophils in bronchoalveolar lavage

**Figure 3:**
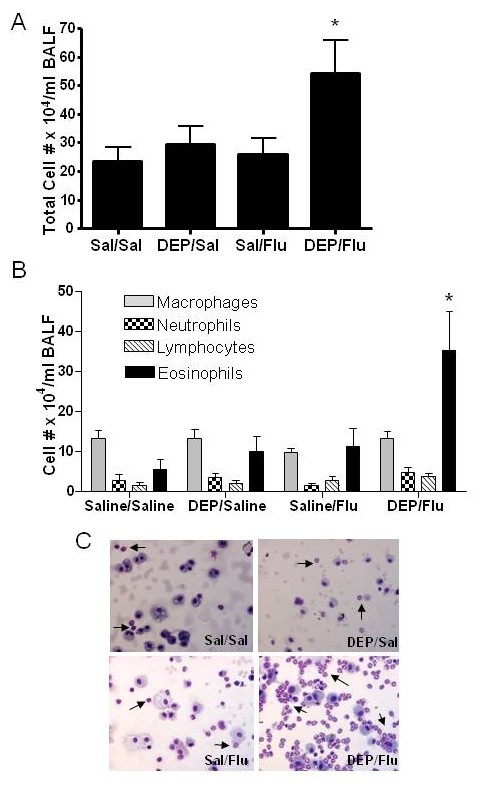
**Exposure to DEP prior to infection with influenza increases BALF eosinophils levels in OVA-sensitized mice**. A.) Total and B.) differential cell count of BAL fluid from OVA-sensitized mice exposed to DEP and/or infected with influenza A. C.) Representative images of BALF cells in obtained from the different experimental groups. *significantly different from Sal/Sal control; p < 0.05.

Total and differential cells counts were assessed in BALF of all four experimental groups. Figure 3A shows that the number of total recoverable cells in the BALF was only significantly enhanced in animals exposed to DEP prior to infection with influenza. Differential cell counts shown in Fig. 3B demonstrate that animals exposed to DEP prior to infection with influenza had significantly higher levels of eosinophils, while the number of any other cell type in the BALF did not significantly differ among the experimental groups. Representative images supporting the large increase of eosinophils in the BALF of animals exposed to DEP prior to infection with influenza (DEP/Flu) are shown in Fig. 3C. However, no changes in cell differentials were observed in peripheral blood leukocytes (Table 1).

**Table 1:** Peripheral blood differential leukocyte count.

	**Monocytic Cells**	**Neutrophils**	**Lymphocytes**	**Eosinophils**
**Sal/Sal**	8.70 +1.08 (n = 5)	59.50+6.05 (n = 5)	31.80+6.88 (n = 5)	6.40+1.76 (n = 5)

**DEP/Sal**	10.40+1.85 (n = 5)	49.90+5.37 (n = 5)	39.70+4.07 (n = 5)	8.50+1.76 (n = 5)

**Sal/Flu**	8.80+1.18 (n = 5)	57.70+3.72 (n = 5)	33.40+4.02 (n = 5)	7.40+1.11 (n = 5)

**DEP/Flu**	8.10+1.50 (n = 5)	54.00+4.77 (n = 5)	37.90+4.64 (n = 5)	6.20+0.82 (n = 5)

### Exposure to DEP prior to infection with influenza increases the number of lung tissue eosinophils

**Figure 4:**
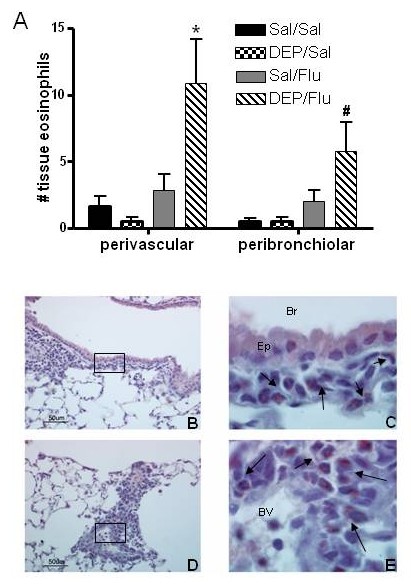
**Exposure to DEP prior to infection with influenza increases tissue eosinophils levels in OVA-sensitized mice**. Lung sections stained for eosinophils using Carbol Chromotrope were analyzed for the presence of perivascular and peribronchial eosinophils. A.) Average numbers of tissue eosinophils located around small airways (150–200 μm) and blood vessels (10–50 μm). B-E.) Representative images from an animal exposed to DEP prior to infection with influenza illustrating the number of infiltrated eosinophils around the airways and blood vessel. Images illustrating peribronchial accumulation of eosinophils (B + C) at 40× (B) and 100× (C) magnification and perivascular accumulation of eosinophils (D + E) at 40× (D) and 100× (E) magnification. Br = bronchi; Ep = epithelium; BV = blood vessel, arrows point to individual eosinophils. *significantly different from Sal/Sal control; p < 0.05.

To further assess eosinophil infiltration into the lung, lung sections were stained using a Carbol Chromotrope to identify eosinophils. Average numbers of tissue eosinophils located around small airways (150–200 μm luminal diameter) and blood vessels (10–50 μm luminal diameter) are shown in Fig. 4A. Similar to eosinophils numbers in BALF, tissue eosinophils were significantly elevated in animals exposed to DEP prior to infection with influenza. Representative images of Carbol Chromotrope stained sections for airways (4B+C) and blood vessels (4D+E) from the DEP/Flu group of animals are shown in Fig. 4B–E.

### Effects of exposure to DEP prior to infection with influenza on markers of eosinophils recruitment

**Figure 5:**
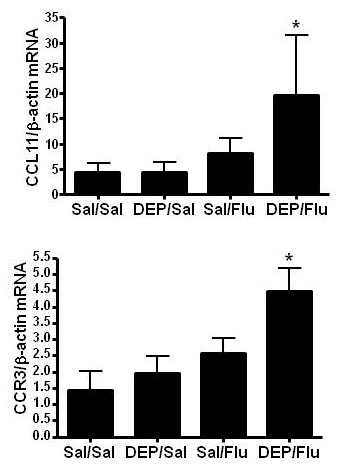
**Exposure to DEP prior to infection with influenza increases markers of eosinophils chemotaxis**. Total mouse lung RNA was analyzed for A.) CCL11 and B.) CCR3 mRNA levels and normalized to β-actin mRNA levels. *significantly different from Sal/Sal control; p < 0.05.

Eosinophil recruitment into the lung is controlled by the release of specific chemokines and expression of their respective receptors on the surface of eosinophils. CCR3 is highly expressed on eosinophils in both humans and mice and mediates chemotaxis and recruitment of eosinophils into the lung [23]. CCR3 binds a number of different chemokines, including eotaxin 1, 2, and 3 (CCL 11, 24, and 26 respectively), as well as MCP-2, 3, and 4 (CCL 8, 7, and 13) and RANTES (CCL5) [23], with CCL11 being more specifically involved in eosinophils recruitment into the lung. Fig. 5A shows that expression of CCL11 was significantly elevated in OVA-sensitized mice exposed to DEP prior to infection with influenza. Similarly, expression of CCR3 was significantly enhanced in the DEP/Flu experimental group as shown in Fig. 5B. Interestingly neither exposure to DEP or infection with influenza alone had any significant effect on the expression of CCL11 or CCR3 in OVA-sensitized mice.

### Effects of exposure to DEP prior to infection with influenza on inflammatory cytokine expression

**Figure 6:**
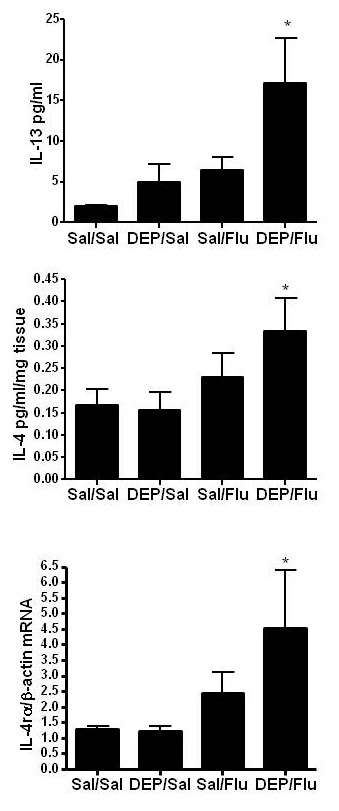
**Exposure to DEP prior to infection with influenza increases markers of Th2-type immune response**. A.) Cell-free BALF was analyzed for IL-13 levels. B.) Whole lung homogenates were analyzed for IL-4 levels. C.) Total RNA isolated from whole lung was analyzed for IL-4rα mRNA levels and normalized to β-actin mRNA levels. *significantly different from Sal/Sal control; p < 0.05.

Next the effects of DEP prior to infection with influenza in OVA-sensitized mice on the expression of inflammatory cytokines were examined, with a specific focus on TH2 cytokines, including IL-13 and IL-4. Fig. 6A shows that the level of IL-13 in BALF was significantly enhanced in animals exposed to DEP prior to infection with influenza. Exposure to DEP or infection with influenza alone resulted in a small, but not statistically significant enhancement of IL-13. Similarly, Fig. 6B shows that levels of IL-4 in lung homogenates (BALF IL-4 levels were below the detection limit; data not shown), were significantly enhanced in animals exposed to DEP prior to infection with influenza. Both IL-4 and IL-13 bind to the alpha chain of the IL-4 receptor (IL-4Rα). Fig. 6C shows that expression of IL-4Rα was significantly increased in animals exposed to DEP prior to infection with influenza. Taken together, these data show that in OVA-sensitized mice, exposure to DEP prior to infection with influenza enhanced the expression of the TH2 cytokines IL-4 and IL-13 and their common receptor IL-4Rα. In addition, a number of other cytokines with known roles in either DEP-induced inflammation or influenza-induced responses were examined in lung homogenates. Table 2 summarizes these results and demonstrates that there were no changes in any groups in the levels of IL-10, MCP-1, and RANTES in lung homogenates. However, IL-1α levels were significantly enhanced in OVA-sensitized animals exposed to DEP prior to infection with influenza.

**Table 2:** Lung homogenate cytokine levels

	**IL-10**	**IL-1α**	**MCP-1**	**RANTES**
**Sal/Sal**	0.59 ± 0.15 (n = 11)	1.57 ± 0.26 (n = 11)	6.83 ± 1.41 (n = 11)	9.84 ± 1.80 (n = 11)

**DEP/Sal**	0.72 ± 0.09 (n = 8)	2.57 ± 0.60 (n = 8)	8.64 ± 1.71 (n = 8)	8.44 ± 1.86 (n = 8)

**Sal/Flu**	0.66 ± 0.11 (n = 10)	2.46 ± 0.37 (n = 11)	8.80 ± 1.99 (n = 11)	8.83 ± 1.25 (n = 11)

**DEP/Flu**	0.73 ± 0.12 (n = 10)	3.99 ± 0.96* (n = 9)	9.08 ± 1.58 (n = 10)	10.70 ± 1.42 (n = 9)

**Cytokine levels in whole lung homogenates**. Whole lung homogenates were analyzed for IL-10, IL-1α, MCP-1, and RANTES. Cytokines levels were normalized to lung tissue weight and expressed as pg/ml/mg tissue. *significantly different from Sal/Sal control; p < 0.05

### Effects of DEP and influenza virus infection on OVA-specific antibody levels

The OVA sensitization and challenge model used here will result in OVA-specific antibody formation. To determine whether exposure to DEP, infection with influenza, or exposure to DEP prior to infection with influenza affected OVA-specific antibody levels, we examined OVA-specific IgG2c, IgE, and IgG1 levels in sera from mice. Table 3 shows that exposure to DEP, infection with influenza, or the combination treatment had no significant effects on OVA-specific IgG2c or IgG1 levels. OVA-specific IgE levels were elevated in animals exposed to DEP alone, influenza alone, and DEP prior to infection with influenza as compared to the control group (Sal/Sal), albeit not statistically significant.

**Table 3:** OVA-specific immunoglobulin levels

	**IgG2c**	**IgE**	**IgG1**
**Sal/Sal**	354.4 ± 108.4 (n = 10)	42973 ± 8680 (n = 11)	384.5 ± 58.54 (n = 10)

**DEP/Sal**	199.3 ± 51.38 (n = 9)	72669 ± 13391 (n = 8)	428.1 ± 30.89 (n = 9)

**Sal/Flu**	128.0 ± 38.94 (n = 9)	79598 ± 13747 (n = 10)	488.6 ± 41.34 (n = 11)

**DEP/Flu**	355.2 ± 131.3 (n = 10)	78654 ± 14977 (n = 10)	434.1 ± 47.33 (n = 10)

### Effects of DEP on influenza viral titers

**Figure 7:**
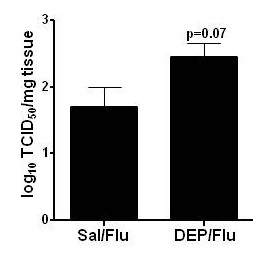
**Effects of exposure to DEP on lung viral titers**. Whole lungs from animals infected with influenza (Sal/Flu and DEP/Flu) obtained 18 hours post-infection were homogenized and analyzed for lung viral titers. Values shown are mean tissue culture infectious dose normalized to tissue weight (TCID_50_/mg tissue).

Next, we examined whether in OVA-sensitized mice, a single instillation of DEP enhanced influenza viral titers in the lung. Influenza viral titers were measured at the time of characterization of allergic inflammation (18 h post-viral inoculation) by using the TCID_50 _assay. As shown in Fig 7, a trend towards greater viral titers was seen in the DEP-treated group (P = 0.07), even at this early-time point post-inoculation.

## Discussion

Respiratory virus infections are by far the greatest risk factor for exacerbation of asthma, especially in children. Considering that concurrent exposures to air pollutants, such as DE, and respiratory viruses are likely, additive or synergistic effects between these exposures in individuals with preexisting allergic airways disease could be of potential public health significance. Although exposure to DE or DEP alone can induce a number of adverse immunological effects including increased inflammatory cytokine production, the effects of DE or DEP have been repeatedly shown to be much greater in conjunction with another immunological stimulus. Our previous work has demonstrated that exposure to DE or DEP increases the susceptibility to influenza virus infections [20,21] Similarly, a number of studies have demonstrated that exposure to DE or DEP increases sensitization to experimental allergens such as ovalbumin [9,24,25]. Our study was designed to test whether and how virus-induced exacerbation of allergic airway inflammation is affected by exposure to oxidant air pollutants. Specifically, we examined how exposure to DEP prior to infection with influenza virus modifies markers of allergic inflammation in a murine model of asthma. Our data shown here demonstrate that in mice with allergic airway disease, exposure to DEP prior to infection with influenza increases the number of lung eosinophils, markers of eosinophils chemotaxis, and TH2 cytokine levels, while exposure to DEP or infection with influenza alone did not. These data indicate that exposure to DEP could potentially sensitize the lung to virus-induced exacerbation of allergic airways inflammation.

Compelling epidemiologic evidence that exposure to oxidant air pollutants can enhance the severity of virus-induced asthma exacerbations comes from a study conducted by Chauhan et al. [22]. This study demonstrated that exposure to the oxidant air pollutant NO_2 _the week before the onset of a respiratory virus infection significantly increased the severity of the resulting asthma exacerbation in a cohort of children. Interestingly, high exposure to NO_2 _the week after the onset of the viral infection was not associated with changes in lower respiratory tract symptoms or peak expiratory flow measurements [22]. These data suggest that exposure to NO_2 _had a priming effect on the symptoms associated with the subsequent naturally acquired viral infection in children with asthma. Our data presented here show similar results in that exposure to DEP or infection with influenza alone did not increase markers of allergic inflammation. Only animals that were exposed to DEP prior to infection with influenza showed significant changes in the number of BALF/lung eosinophils, CCL11, CCR3, or TH2 cytokines, suggesting that exposure to DEP primed the animals for virus-induced exacerbation of allergic inflammation.

We found a trend towards increased viral titers 18 hours post viral inoculation in the DEP-treated groups, at the time we observed enhanced allergic inflammation in the lung. These findings are consistent with previous studies which have demonstrated at later time points that repeated exposures to DE, prior to infection with influenza, increase markers of viral replication in epithelial cells *in vitro *and in mice *in vivo *[20,21]. A potential mechanism for this enhanced viral replication following DE exposure is a reduction in the expression of important antimicrobial defense molecules, a recent observation by our group [21,26]. The potential clinical significance of these findings is highlighted by human studies showing a relationship between TH1/2 immune responses, viral load, and symptom severity in asthmatic subjects experimentally infected with RV [27].

Whether virus-induced exacerbation of asthma is associated with an increase in total viral load or a shift in the location of the infection remains to be determined. For example, a recent study demonstrated that asthmatics can harbor RV infection in the lower airways and that this shift in location of the RV infection is associated with virus-induced exacerbation of asthma [28]. In addition, another study demonstrated that the severity of lower respiratory tract symptoms in subjects diagnosed with a respiratory virus infection was greater in asthmatics, but that these effects were not related to the viral load in the upper respiratory tract [29]. Thus, we cannot rule out the possibility that exposure to DEP can modify the distribution of the viral infection in the lower respiratory tract, and potentiate allergic inflammation by that mechanism.

Recruitment of eosinophils into the lung is regulated by the expression of specific chemokines such as the eotaxins (CCL11 and CCL24 in mice), which are selective agonists for the C-C chemokine receptor 3 (CCR3). Considering the significant influx of tissue and BALF eosinophils, it is likely that the enhanced CCR3 we observed in our study are derived from the increased number of CCR3-expressing cells. In addition to observing increased numbers of CCR3-expressing eosinophils, our data also showed that exposure to DEP prior to infection with influenza increases the expression of CCL11. Cellular sources for CCL11 include endothelial cells, mast cells, fibroblasts, airway epithelial cells, smooth muscle cells, eosinophils, and various other cell types. Its expression can be activated by a number of inflammatory cytokines, such as IL-1, TNF and IFN [30]. In addition, exposure to DE alone has been shown to increase the expression of eotaxin in airway epithelial cells [31] and instillation with DEP alone or in combination with OVA increased lung eotaxin levels [32]. However, our data suggest that in mice sensitized and challenges with OVA, a single exposure to DEP alone is not sufficient to significantly enhance the expression of eotaxin in the lung. Similarly, only mice exposed to DEP prior to infection with influenza showed significant increases in BALF and tissue eosinophils. Potential reasons for these differences include the fact that we used a single instillation of DEP shortly after the last OVA challenge rather than repeated instillations of DEP in combination with OVA challenge [32]. In addition, the type of DEP and its chemical characteristics can significantly affect its ability to potentiate allergic inflammation in OVA-sensitized mice as has recently been demonstrated [33]. The authors of that study demonstrated that BALB/c mice instilled with three chemically distinct DEP samples during the OVA sensitization phase responded differently with regards to potentiation of allergic inflammation. Furthermore, we used C57BL/6 mice in the studies presented here, which are less responsive to OVA-induced allergic airway inflammation as compared to BALB/c mice [34]. Similarly, DEP-induced exacerbation of allergic inflammation differs among mouse strains [35]. Thus, in addition to timing of the DEP exposure in relation to sensitization and/or challenge with OVA, chemical composition of the DEP sample as well as mouse strain are important determinants with regards to DEP-induced enhancement of allergic inflammation.

A number of studies have shown that IL-1 may play a central role during allergic inflammation. For example, administration of an IL-1 receptor antagonist (IL-1ra) decreased lung eotaxin levels and infiltration of eosinophils in OVA-sensitized mice [36]. The authors suggested that IL-1 is necessary for allergen-specific TH2 cell activation and allergic inflammation and that administration of recombinant IL-1ra decreases allergic inflammation either directly by inhibiting the pro-inflammatory activity of IL-1 or indirectly by inhibiting the expression of IL-5 and eotaxin [36]. Our data indicate that in mice exposed to DEP prior to infection with influenza, BALF IL-1 levels were significantly enhanced as compared to the other groups. Thus, it is conceivable that in animals that were exposed to DEP prior to infection with influenza, an increase in IL-1 sets off a cascade culminating in the enhanced expression of TH2 cytokines and accumulation of eosinophils in the lung.

In conclusion, to our knowledge this is the first study providing direct experimental evidence that exposure to air pollutants prior to infection with respiratory virus significantly exacerbates allergic airway inflammation. Since concurrent exposure to air pollutants and infection with respiratory viruses is likely, these findings could be of significant public health implication. This notion is supported by epidemiological studies demonstrating that exposure to oxidant air pollutants prior to the onset of a respiratory virus infection do in fact enhance exacerbation of asthma in a cohort of children [22]. Future studies are necessary to further depict the molecular mechanisms by which exposure to oxidant air pollutants, potentiate virus-induced exacerbation of allergic inflammation.

## Materials and methods

### Animals

Similar to our previous studies using influenza virus infection [37,38] or an ovalbumin sensitization model [39], male C57BL/6 mice 6–8 weeks old were used throughout the study. All experimental procedures were approved by the University of North Carolina IACUC. Based on our previous studies [39] and as outlined in Fig. 1, mice were sensitized on days 1 and 2 by i.p. injection of 100 μl of 1% ovalbumin/alum solution. On days 14, 15, and 16 mice were challenged with aerosolized ovalbumin (1% wt/v) for 30 min/day. A minimum of 5 animals were used for each endpoint.

### Oropharyngeal Aspiration of Diesel Exhaust Particles

Diesel exhaust particles (DEP) were kindly provided by Dr. M. Ian Gilmour and collected as described before [40]. Briefly, a 30-kW (40 hp) four-cylinder Deutz BF4M1008 diesel engine connected to a 22.3-kW Saylor Bell air compressor to provide a load was used to generate the DEP. The engine and compressor were operated at steady state to produce 0.8 m^3^/min of compressed air at 400 kPa. This translates to ~20% of the engine's full-load rating. Emissions from the engine were diluted with filtered air (3:1) to near ambient temperatures (~35°C) and directed to a small baghouse (Dusyex model T6-3.5-9 150 ACFM pyramidal baghouse using a polyester felt bag). Gram quantities of DEP were collected from the baghouse using reverse air pulsing. Once collected, the DEP samples were stored in sealed containers in a refrigerator (~4°C). For the exposure, DEP was suspended in sterile HBSS and sonicated prior to oropharyngeal aspiration of DEP as described before [41]. Briefly, on day 16 approximately 4 hours after the last OVA challenge, animals were anesthetized using vaporized halothane and suspended on their incisors. The tongue was distended and a bolus of either 50 μl HBSS vehicle or 25 μg DEP in 50 μl HBSS was injected onto the oropharynx. Involuntary aspiration was induced by blocking the animal's nares.

### Influenza virus infection

Influenza A/PR/8 (H1N1) was propagated in 10-day-old embryonated hens' eggs. The virus was collected in the allantoic fluid and titered by hemagglutination as described by us before [37,38]. For virus inoculation, mice were anesthetized with an intraperitoneal injection of ketamine (0.022 mg) and xylazine (0.0156 mg) on day 17 (approximately 20 hours after instillation with DEP or HBSS vehicle) and instilled intranasally with 500 pfu of influenza virus in 0.05 mL of PBS.

### Bronchoalveolar Lavage

Approximately 18 h post infection (on day 18), mice from each treatment group were euthanized with sodium pentobarbital and the trachea was exposed, cannulated, and secured with suture thread. The left mainstem bronchus was isolated, clamped with alligator clips after the trachea was cannulated. The right lungs lobes were lavaged 3 times with three volumes of warmed Hanks balanced salt solution (HBSS) (Invitrogen, Grand Island, NY) (35 ml/kg). The resulting lavage was centrifuged (500 × g, 5 min, 4°C) and cell-free lavage fluid was stored at -80°C for cytokine measurement. The pelleted cells were resuspended in 1 ml of RPMI 1640 (Gibco, Carlsbad, CA) containing 2.5% fetal bovine serum (FBS; Gibco, Carlsbad, CA). Total cell counts in the lavage fluid of each mouse were obtained using a hemacytometer. Each sample (100 and 300 μl) was centrifuged in duplicate onto slides using a Cytospin (Shandon, Pittsburgh, PA) and subsequently stained with Diff Quik solution (American Scientific, McGraw Park, PA) for cell differentiation determination, with at least 200 cells counted from each slide. The left lobe was then removed for RNA, protein isolation, or immunohistochemistry.

### Differential Peripheral Blood Leukocyte Count

Blood was collected by cardiac puncture and stored in EDTA-tubes. Each sample was centrifuged onto slides using a Cytospin (Shandon, Pittsburgh, PA) and subsequently stained with Diff Quik solution (American Scientific, McGraw Park, PA) for cell differentiation determination, with at least 200 cells counted from each slide.

### Cytokine Measurements

IL-13 concentrations in cell-free bronchoalveolar lavage (BAL) as well as IL-4, IL-10, IL-1α, MCP-1, and RANTES in lung homogenates were measured using a Cytokine Fluorescent Bead Immunoassay Assay Kits (Beadlyte^®^; Millipore, Billerica, MA). Lung homogenates were prepared by using FastPrep^® ^Lysing Matrix tubes (Millipore). Briefly, snap-frozen lung tissue was homogenized in HBSS using the Lysing Matrix D tubes and the Bio101^® ^FastPrep^® ^system (MP Biomedicals, Solon, OH). Homogenates were centrifuged to clear cellular debris and the cytokine levels were normalized to mg tissue used in the homogenate.

### Real Time PCR

Total RNA was extracted from snap-frozen lung tissue with TRIzol (Invitrogen, Grand Island, NY) as per the supplier's instructions. First strand cDNA synthesis and real-time RT-PCR were performed as previously described [20] using commercially available primers and probes (Applied Biosystems, Foster City, CA).

### Histology

Lung tissue samples were fixed in 4% paraformaldehyde and embedded in paraffin. Five μm thick sections were placed on Superfrost/plus slides (Fisher Scientific) and stained using hematoxylin and eosin (H&E; Richard Allan, Richland, MI) to assess inflammatory infiltrates. To identify tissue eosinophils, sections were stained using a Carbol Chromotrope stain, using Chromotrope 2R (Sigma) dissolved in Phenol. Sections were counterstained using hematoxylin and the slides were evaluated under light microscopy. Five to ten fields of at least 3 animals per experimental group were evaluated for the H&E stain. Tissue eosinophils were enumerated by counting eosinophils surrounding blood vessel and airways using 40× and 100× magnification. Similar to previous reports [42], at least 5 visual fields with blood vessel (diameter 10–50 μm) or bronchioles (diameter 150–200 μm) per animal were evaluated for n = 5 animals per experimental group.

### Quantitation of Viral Titers

Lung viral titers were determined by a modified tissue culture infections dose 50 (TCID_50_) using hemagglutination as an endpoint, as previously described [43]. Briefly, snap frozen lung tissue was weighed and homogenized using homogenized in minimal essential medium using the Lysing Matrix D tubes and the Bio101^® ^FastPrep^® ^system (MP Biomedicals, Solon, OH). Samples were centrifuged at 9000 × *g *for 20 min and the supernatant was serially diluted starting at 1:5 in MEM containing 20 mg/L trypsin. Each diluted supernatant (100 *μ*L) was added, in 6 replicates, to 80% confluent MDCK cells and incubated at 37°C for 72 h. A 0.5% suspension of human O RBC (50 *μ*L) was added to each well and incubated at room temperature for 2 h. Viral titer was expressed as the reciprocal of the highest dilution at which the RBC agglutinated. This value was then normalized to mg tissue of the sample.

### Analysis of OVA-specific immunoglobulin levels

High binding microplates were coated with 100 μl ovalbumin (Sigma) in PBS at 1 mg/ml (IgG1 and IgG2c assays) or 20 mg/ml (IgE assay) at 37°C for 2 hours, followed by incubation with 200 μl/well blocking buffer (1% BSA in PBS) for 1 hour at room temperature. Standard curves for IgG1 and IgG2c were prepared by diluting pooled sera that had previously tested as highly concentrated in OVA-specific IgG1 or IgG2c. In the IgE assay, mouse anti-ovalbumin IgE (AbD Serotec, Raleigh, NC) was used as a standard. One-hundred microliters of each standard or sample dilution was applied to the wells in triplicate and incubated overnight at 4°C and 100 μl of biotinylated anti-Mouse IgG1 (BD Biosciences; 2 ug/ml), biotinylated anti-Mouse IgG2c (Southern Biotechnology; 1:5000), or biotinylated anti-Mouse IgE (BD Biosciences; 4:1000) was added to each well and incubated at room temperature for 30 minutes. Streptavidin-HRP (BD Biosciences; 1:1000), was applied to each well and incubated 30 minutes at room temperature, followed by the addition of TMB substrate (eBioscience). Absorbances were read at 450 nm and concentrations for IgG1 and IgG2c are expressed as arbitrary units.

### Statistical Analysis

Data are shown as mean ± S.E.M. At least 5 animals per experimental group were analyzed, although many endpoints were examined in >5 animals. Data were analyzed using a one-way ANOVA followed by Bonferroni post-hoc test to determine significant differences among the individual groups. A value of p < 0.05 was considered statistically significant.

## Abbreviations

OVA: ovalbumin; DEP: diesel exhaust particles; Flu: influenza; BALF: bronchoalveolar lavage fluid; Sal: Saline; Ig: Immunoglobulin; NO_2_: nitrogen dioxide; IL: interleukin; TNF: tumor necrosis factor; CCL: chemokine (C-C motif) ligand; CCR: C-C chemokine receptor

## Competing interests

The authors declare that they have no competing interests.

## Authors' contributions

IJ conceived of the study, participated in its design and coordination, evaluated the overall study, and drafted the manuscript; PAS carried out the viral infections and assisted in the tissue analysis; WZ carried out the cytokine analysis; LEB carried out the histology analysis; KDC conducted and analyzed the OVA-specific immunoglobulin assays; XH participated in the animal exposures and tissue collection; SLT participated in the design of the study, coordinated the animal exposures, and helped in the tissue collection and drafting of the manuscript. All authors read and approved the final manuscript.
